# IgG4-related disease with massive pericardial effusion diagnosed clinically using FDG-PETCT: a case report

**DOI:** 10.3389/fimmu.2023.1285822

**Published:** 2023-12-22

**Authors:** Qiaofeng Wei, Huili Qi, Hongmei Wei, Xiuhua Wang, Hongju Zhang

**Affiliations:** ^1^ Department of Rheumatology, Zibo Central Hospital, Shandong, Zibo, China; ^2^ Huaqiaocheng Clinic, Zibo Central Hospital, Shandong, Zibo, China; ^3^ Department of Rheumatology and Autoimmunology, The First Affiliated Hospital of Shandong First Medical University, Shandong Provincial Qianfoshan Hospital, Jinan, Shandong, China

**Keywords:** pericardial effusion, IgG4-related disease, FDG-PETCT, prognosis, treatment

## Abstract

**Background:**

IgG4-related disease (IgG4-RD) is a systemic inflammatory disease which involves various organs such as the pancreas, lacrimal gland, salivary gland, retroperitoneum, and so on. These organs can be affected concomitantly. 18-Fluorodeoxyglucose positron emission tomography computed tomography (FDG-PETCT) is a systemic examination which can identify active inflammation and detect multiple organ involvement simultaneously. Pericardial involvement is rare in IgG4-RD, early detection and treatment can greatly improve the prognosis of patients.

**Case summary:**

We reported a 82-year-old female patient referred to our department complaining of chest tightness and abdominal fullness for 8 months and massive pericardial effusion for 2 months. A large amount of pericardial effusion was found during the hospitalization of Gastroenterology. Then she was transferred to cardiology. Although infectious, tuberculous, and neoplastic pericardial effusions were excluded, there was still no diagnosis. The patients were examined by FDG-PETCT which considered IgG4-RD. After coming to our department, the results of the patient’s laboratory tests showed that immunoglobulin subgroup IgG4 was 14.0 g/L. Then we performed a biopsy of the right submandibular gland. Pathological morphology and immunohistochemistry suggested IgG4-RD. Combined with level of IgG4, clinical, pathological and immunohistochemical results, we determined the final diagnosis of IgG4 related diseases. Then we gave glucocorticoid and immunosuppressant treatment. At the end, pericardial effusion was completely absorbed. As prednisone acetate was gradually reduced, no recurrence of the disease has been observed.

**Conclusion:**

Pericardial effusion can be the initial presentation in IgG4-RD. For patients with massive pericardial effusion of unknown cause, early detection of IgG4 is recommended, and PETCT may be helpful for obtaining the diagnosis.

## 1 Introduction

IgG4-related disease (IgG4-RD) is a systemic autoimmune disease with unknown etiology (1–3). The main characteristics of the disease are elevated serum IgG4 concentrations, marked lymphoplasmacytic infiltration, storiform fibrosis, and organ infiltration by IgG4-positive plasma cells. IgG4-RD may be present in a certain percentage of patients with various diseases including autoimmune pancreatitis, Mikuliçz’s disease (MD), Küttner’s tumor (KT), retroperitoneal fibrosis, Riedel’s thyroiditis, hypophysitis, interstitial pneumonitis, interstitial nephritis, prostatitis, lymphadenopathy, inflammatory aortic aneurysm, and inflammatory pseudotumor (4). Although there are some reports about IgG4-RD cases complicated with cardiovascular system, it is rare to have pericardial effusion as the main manifestation (5–7). We herein report a case of IgG4-RD that mainly manifested as massive pericardial effusion, and 18-Fluorodeoxyglucose positron emission tomography computed tomography (FDG-PETCT) first suggested that it was IgG4-RD.

## 2 Case presentation

### 2.1 Chief complaints

An 82-year-old female patient was referred to our department complaining of chest tightness and abdominal fullness for 8 months, with massive pericardial effusion for 2 months.

### 2.2 History of present illness

Eight months prior, the patient developed chest tightness and abdominal distension, which worsened over time. She was admitted to the gastroenterology ward of our hospital. Chest CT showed a large amount of pericardial effusion and a small amount of bilateral pleural effusion. Then, the patient was transferred to the cardiology department for further treatment. Relevant laboratory tests and inspections were performed, but no cause was found. After diuretic treatment, the patient’s symptoms were gradually relieved, and echocardiography showed that pericardial effusion slightly decreased, and she was discharged. The patient underwent an FDG-PETCT examination at Shibo High Tech Hospital, which showed that pericardial effusion might be related to IgG4-RD. The patient was given an over-the-counter diuretic that contained furosemide and spironolactone. Later, the above symptoms reappeared and gradually worsened, and the patient attended our department for further treatment.

### 2.3 History of past illness

She had binocular cataract surgery one year ago.

### 2.4 Physical examination

The patient had a temperature of 36.5°C, a blood pressure of 128/84 mmHg, a pulse of 95 beats per minute, a respiratory rate of 18 breaths per minute, and a finger oxygen saturation of 95%. Cardiac percussion (cardiac dullness) revealed an enlarged area, and a low and dull sound was heard on auscultation. The patient also showed mild pretibial pitting edema. The rest of the physical examination was unremarkable.

### 2.5 Laboratory examinations

In the department of gastroenterology of our hospital, the results of the patient’s laboratory tests showed that the erythrocyte sedimentation rate (ESR) was 67.0 mm/h, D-dimer was 6.42mg/L FEU, brain natriuretic peptide (BNP) was 649ng/ml, total protein was 72.6 g/L, globulin was 35.3g/L, albumin was 35.3 g/L, and immunoglobulin (IgG) was 19.0 g/L. Blood cultures, PPD, T-spot, antinuclear antibody, and extractable nuclear antigen antibody spectrum were all negative. Routine blood tests, routine biochemical tests, thyroid function, tumor markers, coagulation series, pre-transfusion tests, myocardial markers, and routine and urinary tests revealed no significant abnormality.

Some investigations were repeated while the patient was admitted to the rheumatology and immunology department of our hospital. ESR was 29.0 mm/h, D-dimer was 1.86mg/L FEU, BNP was 486ng/ml, total protein was 74.6 g/L, globulin was 35.9g/L, albumin was 38.7 g/L, IgG was 17.2g/L, immunoglobulin subtype 4(IgG4) was 14.0g/L, complement C3, complement C4, C-reactive protein (CRP), and rheumatoid factors were normal. Antinuclear antibody, antineutrophil cytoplasmic antibody, anti-cyclic citrate peptide antibody, and anti-phospholipid antibody were all negative.

### 2.6 Imaging examinations

**Figure 1:**
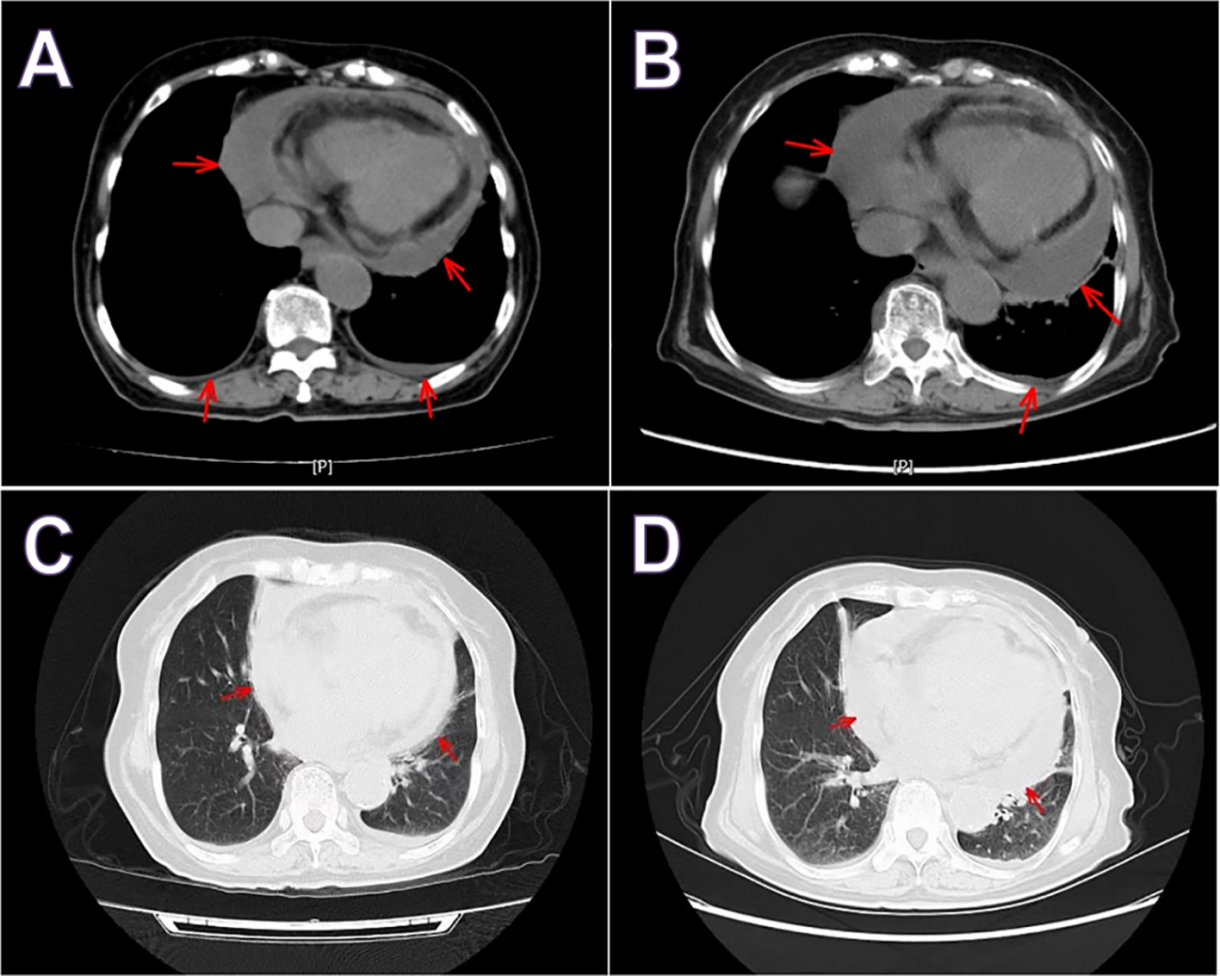
Chest computed tomography hospitalizations in gastroenterology **(A, C)** and rheumatology **(B, D)**. Both showed a large amount of pericardial effusion and a small amount of pleural effusion. Red arrow indicate pericardial effusion.

**Figure 2:**
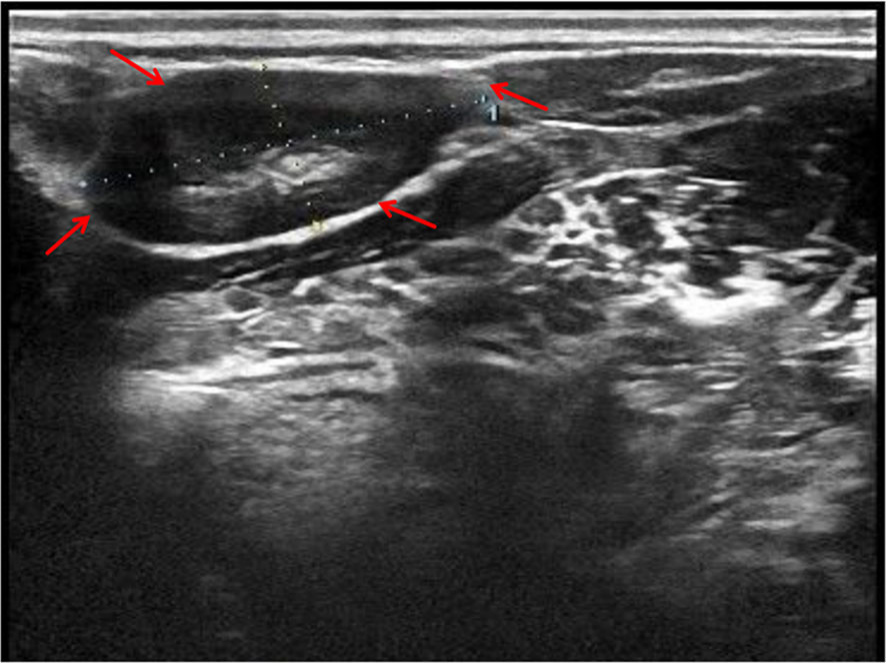
Submandibular gland ultrasound showed that large low echo nodules can be detected, with clear boundaries, less uniform internal echoes, and blood flow signals. Red arrow indicates the submandibular gland nodules.

**Figure 3:**
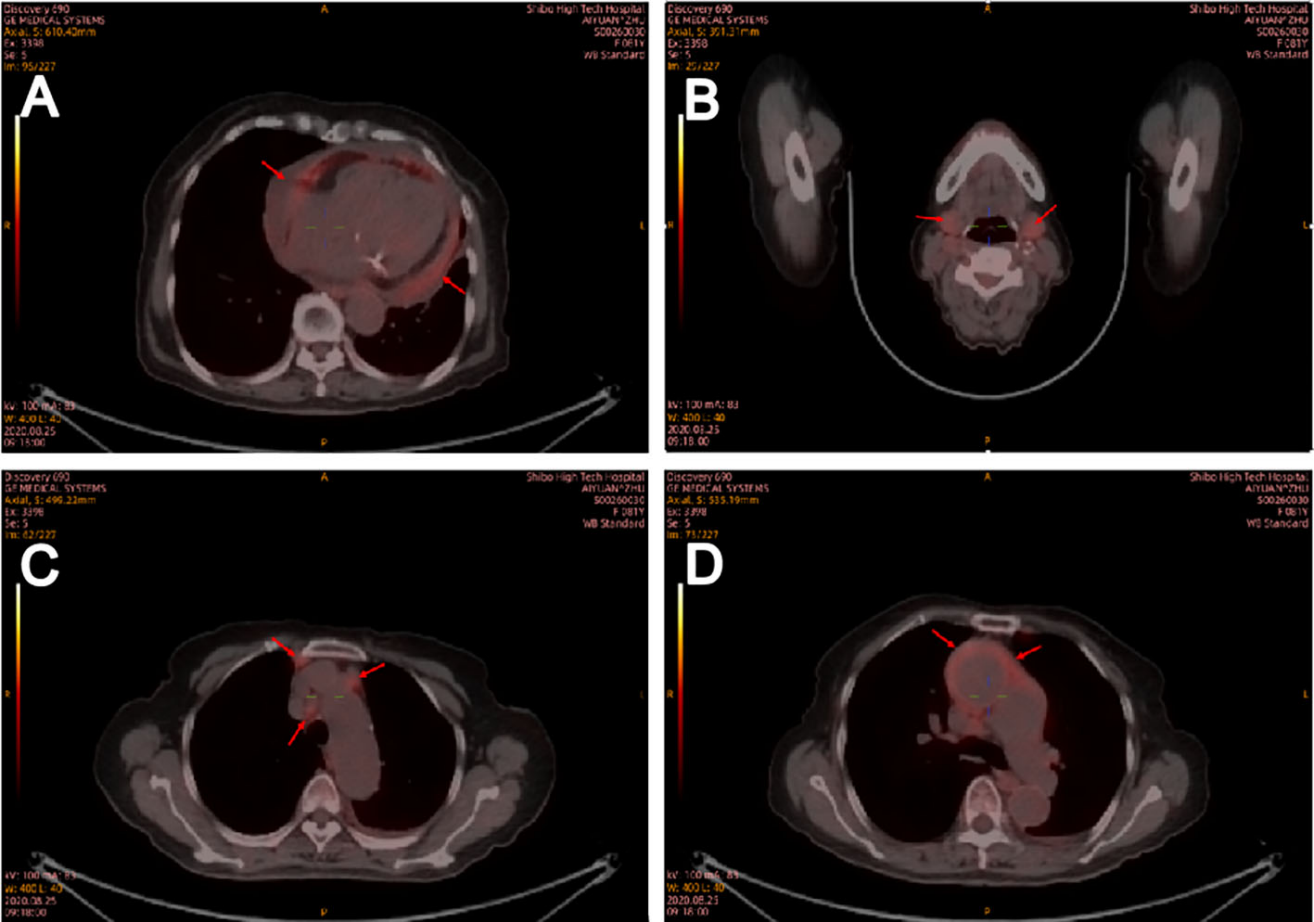
Positron emission tomography computed tomography on August 25, 2020. Positron emission tomography computed tomography showed that medium pericardial effusion which corresponding to an increased area of FDG uptake **(A)**. Bilateral pharyngeal tonsil, submandibular gland **(B)**, small lymph nodes in the right side of sternal bone and mediastinum **(C)** and the wall of ascending aorta **(D)** all showed increased metabolism. Red arrow indicates positron emission tomography (CT) scans showing an increase in FDG uptake area in the central envelope effusion.

In the department of gastroenterology of our hospital, chest computed tomography (CT) showed a large amount of pericardial effusion and a small amount of bilateral pleural effusion, with multiple small nodules in both lungs (**Figures 1A, C**). Echocardiography revealed a large amount of fluid in the pericardial cavity. The depth of the posterior wall of the left ventricle was 16mm, the depth of the anterior wall of the right ventricle was 14mm, the depth of the left ventricle wall was 17mm, and the depth of the right atrial roof was 16mm. Thyroid ultrasound showed multiple thyroid nodules TI-RADS-3, swollen lymph nodes on the right side of the neck. Submandibular gland ultrasound showed that large low echo nodules can be detected in the submandibular gland, with clear boundaries, less uniform internal echoes, and blood flow signals. (**Figure 2**). Abdominal ultrasound displayed no obvious abnormalities. Echocardiography showed moderate fluid accumulation in the pericardial cavity. The depth of the posterior wall of the left ventricle was 11.9mm, and the depth of the left ventricle wall was 14mm. Furthermore, PETCT showed moderate pericardial effusion, which was in accordance with the increased area of FDG uptake, with a maximum SUV of 5.04mm. Bilateral pharyngeal tonsils and submandibular glands manifested symmetrically increased FDG uptake. Moreover, small lymph nodes were observed at the right side of the sternal bone, and the mediastinum and the wall of the ascending aorta all showed increased metabolism (**Figure 3**).

**Figure 4:**
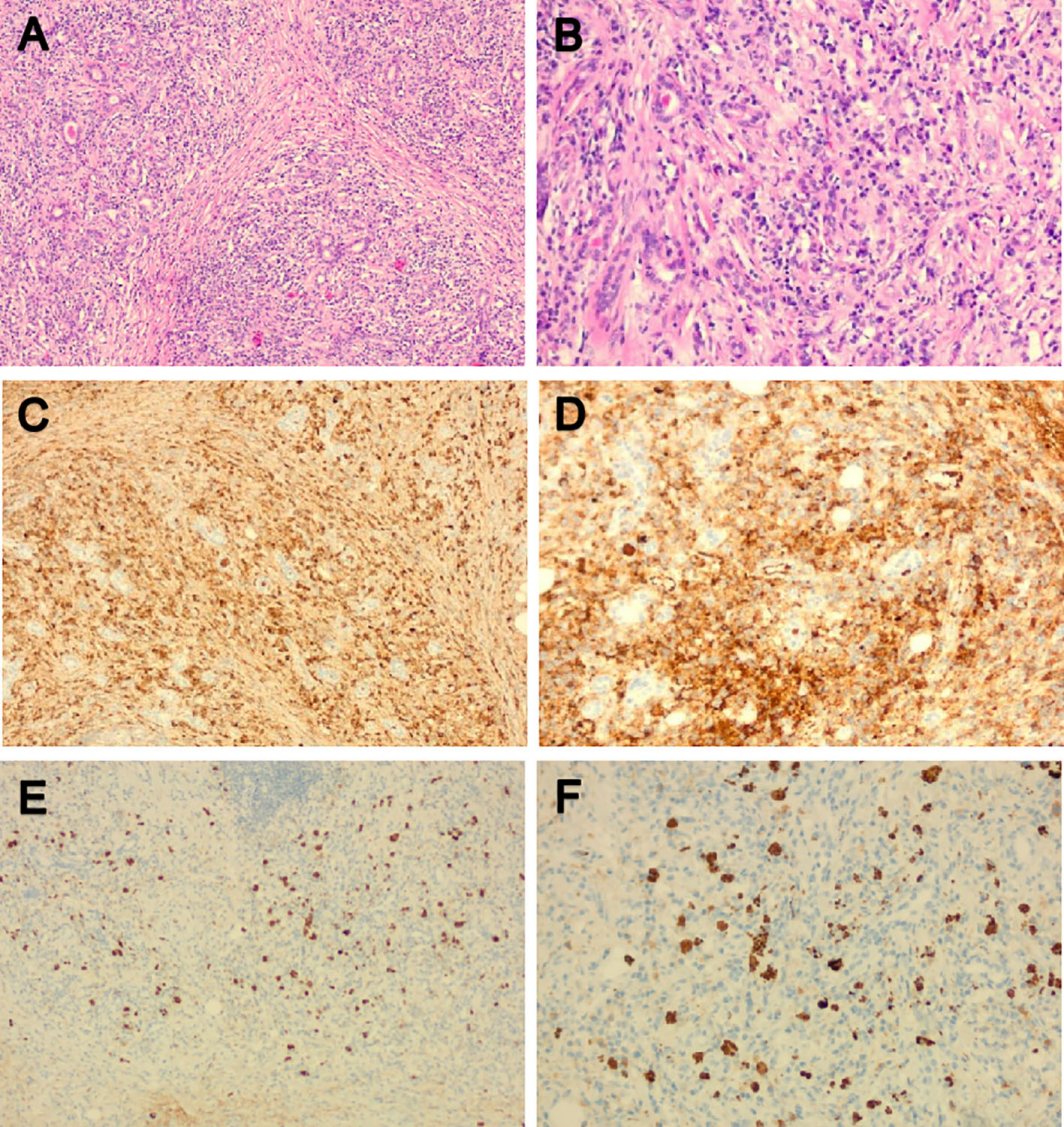
Pathological morphology and immunohistochemistry of the right submandibular gland. **(A, B)** HE showed hyperplasia of interstitial fibrous tissue and lymphoid tissue hyperplasia, formation of lymphoid follicle, infiltration of a large amount of lymphocyte and scattered plasma cells and eosinophils; **(C, D)** Immunoglobulin (Ig) G positive plasma cells could be seen by immunohistochemical staining; **(E, F)** IgG4-positive plasma cells could be seen by immunohistochemical staining; The IgG4/IgG ratio was greater than 40%. HE, Hematoxylin and eosin stain; Ig, Immunoglobulin.

In the rheumatology department, chest CT showed a large amount of pericardial effusion and a small amount of left pleural effusion, multiple small nodules in both lungs, and disseminated inflammation of the middle lobe of the right lung and the lower lobes of both lungs (**Figures 1B, D**). The electrocardiogram showed a sinus rhythm, low T-wave, and increased left atrial load. Echocardiography showed a large amount of fluid in the pericardial cavity. The depth of the posterior wall of the left ventricle was 13mm, the depth of the anterior wall of the right ventricle was 10mm, the depth of the left ventricle wall was 10mm, the depth of the apex was 13mm, and the depth of the right atrial roof was 11mm. Three days after admission, a biopsy of the right submandibular gland was performed (**Figure 4**).

## 3 Final diagnosis

The final diagnosis of the presented case was IgG4-RD.

## 4 Treatment

Treatment with 40 mg methylprednisolone and 1000 mg mycophenolate mofetil per day was initiated. After 2 weeks, pericardial effusion was significantly reduced, ESR, BNP, IgG, and IgG4 were decreased. 13 days after admission, echocardiography showed a small amount of fluid in the pericardial cavity. the depth of the anterior wall of the right ventricle was 4mm, the depth of the apex was 5mm. The patient’s condition gradually improved.

## 5 Outcome and follow-up

She did regular outpatient review after discharge. Echocardiography showed that pericardial effusion was significantly reduced on 15 days after discharge, and completely absorbed on 2 months after discharge. We reduced the amount of prednisone acetate progressively, and the serum levels of IgG4 gradually decreased. No recurrence of pericardial effusion has been observed in the past year. At present, she was treated with 2.5 mg prednisone per day.

## 6 Discussion

IgG4-RD is a systemic autoimmune-mediated fibroinflammatory disorder. In 2001, Hamano et al. (8) reported elevated levels of serum IgG4 in patients with autoimmune sclerosing pancreatitis, and the concept of IgG4-related disease was proposed. Subsequently, Yamamoto et al. (9) identified elevated concentrations of serum IgG4 and the prominent infiltration of IgG4-bearing plasma cells into lacrimal and salivary glands in Mikulicz’s disease (MD), which differed from Sjögren syndrome. Therefore, MD and autoimmune pancreatitis are believed to be related. Then, IgG4-related disease was found to cause inflammation and fibrosis in many other organs (4, 10). In 2012, the first comprehensive clinical diagnostic criteria of IgG4-RD was published (11): ① one or more characteristic diffuse/localized swellings or masses in organs. ② elevated serum IgG4 concentrations (>135mgdl). ③ marked lymphocyte and plasma cell infiltration and fibrosis, infiltration of IgG4+ plasma cells, and IgG4+/IgG+ cell ratio of >40%, and>10 IgG4+ plasma cells/HPF in histopathological examination. Patients who meet the above conditions can be diagnosed as IgG4-RD. For patients with① and ②, or ① and ③, there is a possibility of IgG4-RD. Our case mainly manifested with massive pericardial effusion, showing a serum IgG4 concentration of 14.0g/L. Considering pathological and immunohistochemical results, the patient was diagnosed with IgG4-RD.

Previously, IgG4-RD was believed to be caused by T helper (Th) 2 cells, but this theory has been completely disproved (12, 13). Only IgG4-RD patients with atopic syndrome have circulating Th2 memory cells (14). The discovery of CD4+ cytotoxic T cells in IgG4-RD may be a major breakthrough in pathophysiological research. CD4+T cells are widely distributed in IgG4-RD lesions and constitute the most common cells in the affected tissues. Clonal expansion of CD4+ cytotoxic T cells is found in the peripheral blood and fibrotic lesions of IgG4-RD patients, indicating that these cells play a crucial role in its pathogenesis (15). In addition, these cells produce IL-1, transforming growth factor (TGF-β), and interferon-gamma, which are all important mediators of fibrosis, constituting the main histopathological manifestation of IgG4-RD. The signaling lymphocytic activation module F7(SLAMF7) is expressed on the surface of CD4+ cytotoxic T cells and has not been previously identified on CD4+T cells (16). After glucocorticoid-induced remission of IgG4-RD, such CD4+ cytotoxic T cells were also reduced (17). In addition, Tfh cells mainly exist in and around the germinal centers of lymph nodes and are also increased in the peripheral blood and affected tissues of IgG4-RD patients. Meanwhile, Tfh cells promote the proliferation and differentiation of B cells (18). Follicular regulatory T cells and Tfh cells regulate germinal center formation and B-cell class conversion (19).

IgG4-RD occurs in a variety of organs, including the pancreas, bile duct, retroperitoneum, lacrimal gland, salivary gland, pituitary gland, prostate, lung, heart, kidney, aorta, and thyroid gland. The condition may be misdiagnosed as malignancy, infection, inflammatory disorders, or other immune-mediated disease (20). IgG4-related cardiovascular lesions include aortitis, arteritis, periaortitis, periarteritis, pericarditis, and inflammatory aneurysm (21). To our knowledge, only a few reports have described concurrent IgG4-RD and pericardial effusion. Generally, patients with acute massive pericardial effusion show hemodynamic changes. However, patients with chronic pericardial effusion have relatively stable vital signs (22). Similar to previous case reports, our patient showed no obvious hemodynamic abnormalities, despite a large amount of pericardial effusion. However, this patient’s blood pressure and heart rate were normal and the main symptoms were chest tightness and abdominal fullness. Therefore, pericardial effusion can be the initial presentation in IgG4-RD, and IgG4-associated pericarditis is mostly followed by a chronic course. Nevertheless, the atypical presentation with cardiac and pleural effusion might have led to a misdiagnosis, and failure to administer timely treatment might have resulted in a fatal outcome due to respiratory insufficiency (23).

FDG-PETCT is a commonly used auxiliary diagnostic method for cases with challenging diagnoses. Ozaki et al. revealed that the maximum standardized uptake value (SUV) did not differ significantly in either the early or delayed phase between AIP and pancreatic cancer, but they found multifocal inflammation of the pancreas in AIP, and uptake of FDG by the lacrimal gland, salivary gland, biliary duct, retroperitoneal space, and prostate, which could not be attributed to pancreatic cancer (15). Zhang J et al. (16) reported that FDG-PETCT allowed the detection of the involvement of a greater number of organs compared to conventional evaluations such as physical examination, ultrasonography, and CT. Furthermore, FDG-PETCT demonstrated specific image characteristics and patterns of IgG4-RD. Berti et al. (17) observed that circulating plasmablasts in the serum of IgG4-RD were positively correlated with SUV corrected for partial volume effect (PVC-SUV) and inversely correlated with the total lesion glycolysis (TLG), and found a corresponding reduction in circulating plasmablasts, PVC-SUV, and TLG after immunosuppressive therapies. In the present case, FDG-PETCT indicated that the pericardial effusion of the patient might be due to IgG4-RD. Due to advanced age, the patient refused to perform pericardiocentesis. FDG-PETCT also showed a high uptake of the submandibular gland in the patient, and submandibular gland ultrasound showed that large low echo nodules can be detected, with clear boundaries, less uniform internal echoes, and blood flow signals, which was consistent with diffuse low-echo areas (rock type) multiple low-echoic nodules surrounded by high-echoic linear shadows (cobblestone pattern)reported in previous studies (24, 25). Finally, the patient was diagnosed with IgG4-RD and received the appropriate treatment. Therefore, FDG-PETCT assists in the detection of the extent of organ involvement and the selection of the biopsy site. Moreover, it can also monitor the treatment efficacy in IgG4-RD (18).

Previous literature has also shown that imaging is beneficial in the diagnosis of IgG4-RD-related diseases with pericardial effusion (5, 26, 27). One study reported the case of a 73-year-old Asian man with progressive forced dyspnea, systemic edema, and pericardial effusion. Positron emission tomography and FDG-PETCT revealed an inflammatory focus in the pericardium. Pericardial biopsy revealed IgG4-RD pericarditis (26). Another case report described a 53-year-old woman who attended the hospital due to general discomfort for 6 months, exhibiting significant weight loss and chest discomfort. The patient then underwent cardiac MRI (CMRI) for further evaluation. This shows a marked thickening of the pericardium and active inflammation. The late gadolinium enhancement (LGE) showed extensive circumferential LGE in the pericardium and non-coronary distribution of the left ventricle with subendocardial linear LGE. The final diagnosis was myocardial infarction caused by IgG4-RD (27). The study by Seshika Ratwatte et al. (5) focused on three patients who demonstrated cardiovascular manifestations of IgG4-RD, as well as the shortcomings and importance of early diagnosis. Two cases developed cardiac manifestations before more typical organ systems were affected, leading to delayed diagnosis. Case 1 presented with acute myocardial infarction secondary to IgG4-RD, with CT coronary angiography revealing that the surrounding soft tissue mass involved the anterior descending and left anterior descending branches. In case 2, pericarditis progressed to pericardium contraction due to IgG4-RD. CT coronary angiography showed significant pericardium thickening, but no obstructive coronary artery stenosis or coronary artery wall thickening. However, CMRI showed pericardium thickening and increased T2-weighted imaging signals, suggesting active inflammation with edema. PET imaging indicated pericardial uptake. Collectively, these cases suggest that identifying the cardiac manifestations of IgG4-RD on cardiac imaging can enhance clinical suspicion and facilitate a definitive diagnosis.

The goal of treatment is to control inflammation, achieve and maintain remission, and protect organ function (28). To date, glucocorticoids remain the accepted first-line drug for the treatment of IgG4-RD, which can be used in the induced remission and maintenance phases of the disease. Usually, the treatment with glucocorticoids has a rapid effect and can be improved within a few days to a few weeks. The effective rate of the hormone is more than 90% (29). The specific dosage of the hormone should be individualized and can be adjusted according to age and condition. Gradually decrease to the minimum maintenance dose after disease control. Due to the high recurrence rate of IgG4-RD after discontinuation, it is necessary for doctors to make a long-term maintenance or discontinuation plan according to the situation of each patient (30).

In recent years, traditional immunosuppressants have been increasingly used in the treatment of IgG4-RD as hormone-reducing agents combined with glucocorticoids. Many studies (31) have shown that hormone combined immunosuppressive therapy is more effective than glucocortixxcoids alone in controlling disease and reducing recurrence in IgG4-RD patients. A combination of hormones and immunosuppressants is recommended when the disease is not adequately controlled by hormone therapy alone and glucocorticoid side effects are evident. Among them, mycophenolate and azathioprine are the most widely used in clinical practice. Because of the slow onset of traditional immunosuppressants, immunosuppressant therapy alone is not recommended in patients with acute activity. For patients who have failed conventional therapy, relapsed during hormone reduction, and have hormone resistance or intolerance, rituximab may be considered, but care should be taken to prevent infection after the use of this drug.

Seshika Ratwatte reported two cases of IgG4-RD with pericardial effusium, one of which was treated with glucocorticoid and azathioprine. The other case was treated with glucocorticoid and rituximab, and the effect was good (27). A case of IgG4-RD woman with pericardial effusion, in which rituximab alone also responded favorably (5). For this patient, the patient is old and long-term use of glucocorticoids is prone to side effects such as hypertension, diabetes and osteoporosis, and the combination of immunosuppressants is helpful to stabilize the condition when hormone reduction is achieved. Considering the high price of rituximab, rituximab was not selected in the treatment of this patient. Mycophenolate was used to control the disease.

## 7 Conclusion

We herein reported a case who mainly manifested as massive pericardical effusion, and FDG-PETCT imaging revealed FDG accumulation in the pericardium and pericardial fluid, then suggested IgG4-RD. When the patient referred to our department we found IgG4 markedly elevated, and treatment with 40 mg methylprednisolone and 1000 mg mycophenolate mofetil per day was initiated. Finally, pericardial effusion was completely absorbed. For patients with massive pericardial effusion of unknown cause, early detection of IgG4 is recommended, and FDG-PETCT may be helpful for obtaining the diagnosis. The patient is currently still being followed up.

## Data availability statement

The original contributions presented in the study are included in the article/supplementary material. Further inquiries can be directed to the corresponding author.

## Ethics statement

The studies involving humans were approved by Zibo Central Hospital Medical Ethics Expert Committee. The studies were conducted in accordance with the local legislation and institutional requirements. Written informed consent for participation in this study was provided by the participants’ legal guardians/next of kin. Written informed consent was obtained from the individual(s), and minor(s)’ legal guardian/next of kin, for the publication of any potentially identifiable images or data included in this article.

## Author contributions

QW: Formal Analysis, Investigation, Methodology, Resources, Writing – review & editing. HQ: Software, Supervision, Validation, Writing – original draft. HW: Formal Analysis, Software, Supervision, Validation, Writing – original draft. XW: Conceptualization, Methodology, Project administration, Writing – review & editing. HZ: Conceptualization, Data curation, Funding acquisition, Validation, Visualization, Writing – original draft.
